# The discourses on induced abortion in Ugandan daily newspapers: a discourse analysis

**DOI:** 10.1186/s12978-015-0049-0

**Published:** 2015-06-25

**Authors:** Sofia Larsson, Miriam Eliasson, Marie Klingberg Allvin, Elisabeth Faxelid, Lynn Atuyambe, Sara Fritzell

**Affiliations:** Department of Public Health Sciences, Karolinska Institutet, SE-171 29 Solna, Sweden; School of Health and Social Sciences, Dalarna University, 791 88 Falun, Sweden; Department of Women’s and Children’s Health, Karolinska Institutet, SE-171 29 Solna, Sweden; Department of Community Health and Behavioural Sciences, Makerere University School of Public Health, P.O.Box 7072, Kampala, Uganda; The Swedish Red Cross University College, Box 55676, SE-102 15 Stockholm, Sweden

**Keywords:** Abortion, Discourse, Gender, Girls, Health, Law, Pregnancy, Religion, Stigma, Uganda, Women

## Abstract

**Background:**

Ugandan law prohibits abortion under all circumstances except where there is a risk for the woman’s life. However, it has been estimated that over 250 000 illegal abortions are being performed in the country yearly. Many of these abortions are carried out under unsafe conditions, being one of the most common reasons behind the nearly 5000 maternal deaths per year in Uganda. Little research has been conducted in relation to societal views on abortion within the Ugandan society. This study aims to analyze the discourse on abortion as expressed in the two main daily Ugandan newspapers.

**Method:**

The conceptual content of 59 articles on abortion between years 2006–2012, from the two main daily English-speaking newspapers in Uganda, was studied using principles from critical discourse analysis.

**Results:**

A religious discourse and a human rights discourse, together with medical and legal sub discourses frame the subject of abortion in Uganda, with consequences for who is portrayed as a victim and who is to blame for abortions taking place. It shows the strong presence of the Catholic Church within the medial debate on abortion. The results also demonstrate the absence of medial statements related to abortion made by political stakeholders.

**Conclusions:**

The Catholic Church has a strong position within the Ugandan society and their stance on abortion tends to have great influence on the way other actors and their activities are presented within the media, as well as how stakeholders choose to convey their message, or choose not to publicly debate the issue in question at all. To decrease the number of maternal deaths, we highlight the need for a more inclusive and varied debate that problematizes the current situation, especially from a gender perspective.

## Background

It is estimated that around 46,000 women die every year because of unsafe abortions [1] and that as many as 5 million are either permanently or temporarily disabled because of abortion related complications [2]. In addition, there are psychological responses that are more difficult to assess than physical detriments [3]. Some reasons behind women’s choice to terminate a pregnancy are related to factors such as being very young and wishing to postpone childbirth, relationship problems, economic reasons, rape and/or social stigma related to being pregnant while unmarried [3]. These are life circumstances influenced by gender inequalities and power relations between women and men in the particular societies [3].

Consequences of an unwanted birth can imply being forced to quit school, having to face rejection from one’s family and community, and in some societies even being forced to marry or experiencing serious physical harm. [4, 5] Of the estimated 42 million abortions that are performed annually worldwide, 22 million are carried out under safe conditions whereas the other 20 million are conducted under circumstances where the woman’s health is at serious risk [6].

In many settings there are conflicting views on induced abortion. This ambivalence is often shaped by gender roles as well as contextualized by definitions of what responsible sexual and reproductive behavior entails [7]. In countries where strong conservative religious forces hold social as well as political power, also the media are influenced by them, helping to shape the stigma of abortion [8]. Stigma related to conditions such as HIV/AIDS has been theorized and studied empirically, but stigma related to induced abortion has been studied much less [9]. However, it has been hypothesized that abortion related stigma is negatively affecting women’s mental health as well as leading to cognitive and emotional implications of concealment [10]. This is also something that might affect a woman’s willingness to disclose her abortion intentions and/or experiences to partners, parents, friends or health care providers, which further puts her health and future fertility at risk [10]. Abortion stigma manifests itself on multiple levels, from individual to systematic levels, and it is tied to other forms of repressions such as socio-economic inequality, sexism, and racism [11]. This may not only influence individuals’ physical and mental health and disclosure decisions, but also lead to discrimination [12]. In some countries, such as South Africa, abortion is widely available but still socially proscribed. In other countries it is a clandestine practice which is not accessible, resulting in negative consequences for women [13]. Literature reviews have concluded that women are often met with suspicion or even hostility after having had an induced abortion. An example of this is health care providers’ penal attitudes toward women who seek medical care because of post-abortion complications after having undergone unsafe abortion procedures, frequently manifested as verbal but also physical abuse [14].

Uganda’s reproductive health indicators are poor. For instance, the maternal mortality ratio is 310/100,000 live births, or almost 5000 women dying annually of pregnancy-related causes [15]. The total fertility rate is 6.2 children per woman [16]. In Uganda, only 17 % of all women of reproductive age, and 18 % of married women, utilize modern contraception [17]. In addition to limited access because of sporadic stocks of contraceptives, many providers of contraceptives do not have sufficient knowledge about long-acting methods, emergency contraceptives and barrier methods apart from condoms. [18] Close to half of the 1.4 million annual pregnancies occurring in Uganda are unwanted [17]. Unwanted pregnancies have been strongly linked to unsafe abortions, constituting nearly one third of maternal deaths among the country’s young people [19, 20]. Ugandan law permits abortion under certain conditions, but laws and policies on abortion are imprecise and often interpreted incoherently. This makes it difficult for women as well as the medical community to comprehend what is legally permitted [21]. Because interpretations of the law are unclear, medical providers might be hesitant to perform an abortion for fear of legal consequences. A national estimate of abortion incidence in Uganda reported an annual abortion rate of 53 abortions in every 1000 women, which is much higher than the average rate for Eastern Africa (36 abortions per 1000 women) [22]. It has been estimated that about 297,000 illegal abortions are performed yearly in Uganda [19]. A large proportion of these abortions are conducted under unsafe conditions by people without medical training, resulting in nearly 85,000 women treated annually for abortion related complications [19]. The average cost to the Ugandan healthcare system of treating complications from unsafe abortion was nearly US$130 per patient in 2009 and post abortion care is estimated to cost nearly $14 million annually. Most costs of post abortion care arise from treating incomplete abortions and a substantial proportion is spent treating more serious complications, such as sepsis and perforations [23].

Eighty percent of the Ugandan population is Christian, of which almost half belong to the Catholic Church. The Catholic Church has no official political power in Uganda, but a strong influential control [24]. An example of the faith community’s impact on the political arena in Uganda regarding abortion was manifested a decade ago when a set of teachers’ manuals containing a chapter on safer sex to avoid abortions was introduced by the government. These had to be withdrawn from the curriculum within a year after having been strongly criticized by conservative and religious advocacy groups. Since then Uganda’s official position on sex education remains undefined [25]. A resolution of the Ugandan Parliament making safe motherhood a priority was, however, introduced in 2006 and endorsed by all parties. Efforts to mitigate maternal mortality were also specifically funded in Uganda’s budget for the first time [26].

Some policy makers in Uganda have shown to be in favor of more liberal abortion laws but so far few have articulated this in public. Moreover, few Ugandan lawyers are willing to launch a constitutional challenge against the anti-abortion laws and perhaps even fewer women willing to be involved in a test case, i.e., a judiciary case which outcome is likely to set a precedent or test the constitutionality of a statute [27]. In countries such as Uganda, the debate on abortion from a legislative perspective has yet to begin, even though legal human rights experts acknowledge the need for reform. Some hope that a debate could be triggered as a senior United Nations official openly called for the decriminalization of abortion in 2011. However, it is unlikely that such debate would bring any immediate reforms [27].

In order to better understand what values surround the subject of induced abortion and abortion legislation in Uganda, it is vital to study existing societal discourses on the subject in the country. As journalistic routines, norms and conventions create hegemonic discourse around political issues, it is vital to study how for example mainstream news media represent movements and their causes over time [8].

### Theoretical starting point

Social constructionism suggests that things taken for granted such as laws and customs instead are ‘perspectives’ that can be challenged. They could be regarded as institutionalized perceptions suited to particular groups in society. According to this viewpoint social problems should not be seen as objective conditions, but rather as part of social processes in a wider sense [28]. In critical discourse analysis discourses are defined as diverse representations of social life, which are viewed as inherently positioned [29]. Differently positioned actors perceive and represent social life in different ways. These discourses are ordered and they also interact. One aspect of this ordering is dominance; acknowledging that some ways of making statements are dominant, while others are marginal [29]. The presence or absence of certain views, angles and wordings in a text help to explain prevailing social climates and different power relations [29].

This study begins with a social problem, the common unwanted pregnancies among Ugandan women and that they lead to many, unsafely performed, abortions. We seek to comprehend how the problem of abortion is represented, what assumptions underlie these representations and what consequences these representations have, in order to contribute to a more thorough understanding of abortion stigma in the Ugandan context.

### Aim

The aim is to understand societal views on induced abortion in Uganda, through analyzing the discourses on abortion as expressed in the country’s two main daily English-speaking newspapers.

## Methods and materials

The data was collected through a systematic online search of articles from a six year period (January 1, 2007 to December 31, 2012) published on the websites of the two biggest Ugandan newspapers’ *New Vision* and *Daily Monitor*. The time period was chosen in order to obtain the most recent articles and at the same time keep the amount of material on a manageable level. The newspapers represent one state owned and one independent source of news in English, both stating that they offer independent daily news. These newspapers are read by the English literate part of the population, rather than a majority of Ugandans. Nevertheless, the readers constitute a group with power to influence the societal discourse among the broader population through their professional and social status. Thus, the choice of material is expected to give a good span of prevalent public stands on the issue in question.

“Abortion” was the only word used when searching for relevant articles on respective website’s search engine. On both search engines all categories of articles (such as *news*, *culture*, *national* and *world* etc.) were included. From *New Vision* 45 articles containing the word abortion were identified and from *Daily Monitor* 96 articles were found. Out of these articles the ones expressing views or a position on abortion related issues and those dealing with abortion from a legislative perspective were singled out and included in the study. The excluded articles were articles merely mentioning abortion, while focusing on other topics, such as articles using the word abortion when referring to the termination of something other than a pregnancy. This meant that a total of 59 articles were analyzed, 19 from *New Vision* and 40 from the *Daily Monitor*.

The analysis is inspired by Fairclough’s analytical framework for critical discourse analysis [28] and considering the large size of the material, the goal of the study has been to find a few dominant themes that represent the articles’ textual contents. This was done in order to obtain a general picture of how abortion is portrayed within the chosen media. After reading through all the articles in order to get an overall view of the material, the text of each article was analyzed paragraph by paragraph. Relevant units were thereafter identified and coded in order to compare and categorize them. Examples of such codes include “religion” and “real life stories”. These were then examined to understand assumptions and values within particular discourses. Relationships, including points of conflict, as well as subject positions and counter positions were identified. Also, attention was paid to silences in the texts, that is, what is not said and whose voices are missing from the representations. Quotes that were especially informative, typical or illustrating opposing views were chosen in order to demonstrate prevalent standpoints on the issue of abortion in Uganda. For ethical reasons, names and identifying titles have been removed from the citations. After the initial coding had been conducted by the first and last authors, the interpretation of the data was continuously discussed and re-evaluated by the members of the research team in order to increase credibility.

The researchers constituted a multidisciplinary team, with expertise in public health, political science, cultural anthropology and midwifery. Three of the researchers have previous experience of conducting reproductive health research in Uganda.

There are no ethical issues involved in this study as the examined articles are already published material.

## Results

Through adopting an approach which focuses the analysis on presuppositions, assumptions and effects of different views [27], two main discourses framing the issue of induced abortion were found among the examined articles. The two main identified discourses were equally strong in both newspapers and since no significant difference existed in the way abortion tended to be described the material is referred to as a whole.

A prominent discourse described in the first section of the results, is a religious discourse, emphasizing the sanctity of life of the fetus. This discourse is mainly put forward by representatives of the Catholic Church, stating that life begins at conception and that abortion is murder.

A second identified discourse focuses on human rights aspects, namely the right to life of the unborn child on the one hand and on the other hand women’s rights to health. This discourse is supported by argumentation from legal and medical sub-discourses.

The last section of the results highlights that no matter which discourse is being articulated, it has consequences for how women undergoing abortions are portrayed. On one side stands the reasoning that women are victims as a result of the strict abortion legislation as it forces them to undergo illegal and unsafe abortions. On the other stands the argument that the danger lies in that many Ugandan women break the law by performing abortion.

### Sanctity of life

A salient discourse on abortion is the religious discourse of the sanctity of life of the fetus. This argumentation is put forward by the Catholic Church, which has a strong position in the Ugandan society. Several of the examined articles emphasize statements by religious leaders and representatives, such as bishops, and “pro-life” activists from Catholic movements. These statements condemn abortion, referring to it as ‘murder’ and ‘an evil act’, in addition to arguing that it is not permitted according to the Bible. A central assumption in this discourse is that life begins at conception and that there exist no circumstances that can justify abortion. An example of such recurrent statements is the excerpt below.

*Bishop (…) also condemned abortion as an evil act which the Church is against even when some people have come up to defend it in circumstances they think are justified. He said the Bible is very clear on who has authority over life and has not indicated for which abortion could be permitted* [30].

Even women who have become pregnant through rape ought to keep their babies according to so called pro-lifers. Such argumentation is found in the excerpt below, where this is framed as being in the interest of the woman’s well-being.

*Some women activists, prominent lawyers, opinion leaders and even policy makers tend to manipulate rape stories in pursuance of decriminalising abortion. They castigate the church and pro-lifers as being insensitive and unloving towards raped women. (…) the enduring of the pregnancy and the joy of mother care is the right therapy for rape. The woman learns to accept the baby as her own even more than that of its rapist father. The baby will learn to appreciate the heroism of its mother* [31].

Instead of putting the focus on the negative impacts experienced by raped women, the “pro-lifers” tend to focus their argumentation on the healing power of motherhood, i.e., having their babies will make these women feel stronger and happy again, such as in the quote above. These feelings will further be enhanced by the future gratitude received from the child. Here men’s role and responsibility is not problematized at all and moral language is frequently used to frame the problem of unwanted pregnancies, which tends to be defined as “not to nurture is the problem”.

In addition to stating that abortion equals murder, religious representatives urge girls and young women to restrain from being sexually active in order to avoid getting pregnant with a baby that they might feel incapable of taking care of.

*(A Catholic bishop) has said that abortion is ‘real murder’ and that it should be strongly condemned. He urged women, particularly young girls in schools to stop engaging in sexual activities which could result into several complications including cases of unplanned-for pregnancies and abortion…“The time is now for school girls to stop practicing abortion, since life begins at conception. Aborting is the act of taking human life that has been conceived in a woman’s womb, so it is akin to murder. It is also a direct defiance to God’s accepted idea of the sanctity of human life,” he said* [32].

This discourse rests on a moral argumentation where the problem is framed as girls’ sexuality. Argumentation along the same line, urging young men to restrain from sexual activity, cannot be found within the examined articles. Thus the responsibility for unwanted pregnancies is solely put on the young women.

### The rights of the unborn child or the rights of the pregnant woman?

Another significant discourse on abortion revolves around human rights, on the one hand the right to life of the unborn child and on the other the pregnant woman’s right to health and life. The strive for a liberalization of the abortion legislation in Uganda is supported by human rights- and legal arguments, as well as medical arguments, focusing on women’s health.

While there are no statements made by Ugandan political stakeholders on the issue of induced abortion, health care professionals and activists call for access to safe abortion as part of improved health care in order to promote women’s rights to life and health. Several of the examined articles point out how strict abortion laws affect the health of Ugandan women negatively. Furthermore, it is argued that the current law stigmatizes abortion, leading to increasing numbers of unsafe backstreet abortions:

*If abortion is legalised, many lives of mothers will be saved, a health specialist has said. Dr (…), a resident mentor at the Mulago School of Public Health, said there are over 6000 deaths every year resulting from unsafe abortions which mainly result from stigma* [33].

Moreover, a feature of the rights discourse is that statistics are used to demonstrate how maternal deaths (and costs of treating complications from unsafe abortions) have decreased in other countries as their abortion laws have been changed toward a liberalization;

*Unsafe abortions are common in other countries in sub-Saharan Africa. In South Africa where medical abortion was legalized since 1996, maternal deaths associated with unsafe abortion dropped from 425 recorded in 1994 to less than 30 from 1998 to date* [34].

Also, references are made to international treaties such as the Maputo protocol (The Protocol to the African Charter on Human and Peoples’ Rights on the Rights of Women in Africa, which was adopted by the African Union in 2003 and ratified by Uganda in 2010). The Protocol calls for women’s control over their own reproductive health and example of statement referring to it is shown below.

*Medical practitioners suggest that if the country is to curb this growing evil [maternal deaths], abortion should be legalised. “The fact that people think it’s illegal, they do it in hiding which is unprofessional while others run to traditional herbalists,” Dr (…) said. (…) Dr (…) said for Uganda to overcome the above, it needs to put into practice the Maputo Protocol which is signed with other 52 African Union states. “This agreement advocates licensing abortion and specifies under which conditions a mother should have an abortion. In the long run, it aims at saving the mother’s life,” he said* [33].

At the same time the Maputo Protocol is used also by those who are not in favor of changed abortion laws, such as in the example below, saying that it is a “dangerous document” aiming at destroying African families and societies, as well as neglecting the rights of the unborn child. One argument behind this reasoning is that more Africans are needed in order for the region to become more powerful and not so easily pushed-over by dominant nations outside the continent. It is argued by “pro-life” activists, such as members of the American based Catholic organization *Human Life International,* which is dedicated to “protect human life from contraception to natural death”, that new laws should not be imposed on African countries by the West.

*The coordinator of HLI (Human Life International) in Africa, (…), described the Maputo Protocol, which calls for the legalisation of abortion, as a dangerous document aimed at destroying African families and population. “Our first task is to help promote the culture of life. The Maputo Protocol commands the legalisation of abortion, promotion of contraceptives. This is wrong,” he said* [35].

The representative of the anti-abortion organization above refers to a “culture of life” as an argument against a liberalized abortion law, framing this as an issue oimportance for African interests.

Other examples where viewpoints on the abortion legislation are put forward include articles about individual judiciary cases describing how women, health workers and others have been jailed, arrested or sentenced for undergoing/procuring an abortion, such as in the following article:

*A clinical officer attached to Tororo general hospital has been arrested for allegedly conducting an illegal abortion (….) following a formal complaint lodged to police by (…) the secretary general of Human Life International, a US based organization spear heading the fight against abortion and artificial birth control methods. ‘We cannot allow this kind of practice to continue anymore, we have lost one life and we shall not allow these girls life to follow’ (…) said.” The organization has so far rescued at least 56 girls and women from abortion from various parts of the country in the past five months of which 20 of them gave birth to healthy bouncing babies* [36].

The girls and women are portrayed as needing “rescue” and their own capabilities and rights, to judge if they are capable of bringing up a child in their circumstances, are rejected. The health professional is the criminal, earning a report to the police by the “pro-life” organization.

The birth of a baby is seen as the endpoint and solution to the problem, as the mothers then are blessed with the gift of motherhood. There is no mention of the women’s financial and social circumstances, nor their mental and physical health and how these factors would be affected by the responsibility of raising a child on their own, other than that they will be healed by having their babies.

### Victim or criminal?

Which discourse is being articulated, and in what way, has consequences as to whether the woman undergoing or considering an abortion is portrayed as a victim or as a criminal. On the one hand exists the reasoning that women are victims as a result of the strict abortion legislation as it forces them to undergo illegal and unsafe abortions. On the other hand it is argued that the danger lies in that many Ugandan women, and personnel assisting them, break the law by doing an abortion, i.e., committing murder.

Articles seemingly aimed at demonstrating that abortion is a wrongful act per se, tend to use stories that are told by women who have undergone unsafe abortions and who refer to the act as something they regret. Abortion is portrayed as a traumatic event for the woman resulting in physical complications as well as psychological and emotional agonies.

*Along with the innocent babies, 110,000 women die while procuring abortions or from related complications. One woman narrated her painful ordeal (…) My only option was to abort but the question was how to do it. (…) While in the theatre lying on the hospital bed like I was going to give birth, the doctor inserted some nut like instrument into my privates. Feeling every bit of what he was doing with tears stream out of my eyes (…) I recall the doctor seemed to be drilling a hole into a piece of wood. He kept telling me the operation was going to take about five minutes when I first visited. But no, it was hours. To make matters worse, he kept mocking me, telling me to relax and feel good as he also asked the whereabouts of my boyfriend. (…) Unconsciously, I got off the bed, crawled to the bathroom to empty my stomach and then took a 20 min nap. On gaining my consciousness, the pain simply intensified and I passed out again for like one and half hours. (…) I was glad I had survived the whole process and the truth was just with me and the doctor. But this act has kept haunting me for life, and as I tell this story it’s one thing I would not wish another person to go through. The pain I endured was enough to kill ten horses* [37].

In this narrative, the suffering of the woman who has undergone the abortion is in focus. The suffering is represented as being caused by having committed something wrongful resulting in life-long agony (“this act has kept haunting me for life”). The physician is portrayed as a villain; he lies, causes pain and disrespects the patient.

Argumentation in favor of liberalized abortion laws uses real life stories of individuals less frequently. However, below is an example of such that highlights the perceived negative impacts of a religious discourse.

*The whole thing reminded me of another dear friend who had to terminate her pregnancy at six months because the father of the baby was not responsible enough – and her own father would have ‘killed her if he’d found out. (…) I met this deeply religious woman who, in our conversation, said that women who aborted foetuses deserved to go to hell. (…) Certain people do not seem to understand abortion for what it really is. (…) That is why I say that in the real world, if a woman cannot keep a foetus, she should not be forced to keep one. And this should not be debated by anyone who has never, ever undergone the torture of terminating a pregnancy!* [38].

More frequent among the articles containing argumentation in favor of liberalized abortion legislation are statements made by professionals coming in contact with women who have gone through an unwanted pregnancy since they feared going through an abortion.

*“People are living in very difficult circumstances that force mothers to abandon their children thinking that maybe they will get people to save them. The fear of abortion is what makes them keep the pregnancy,” said Ms […], an officer at the police Child and Family Protections Unit* [39].

Thus, the debate content of these articles is rather polemic. On the one hand it focuses on how abortion is the termination of life and that it is such a traumatic event for the woman undergoing it that it should be avoided at all costs, on the other hand it questions what kind of life girls and women are given in Uganda when so many see themselves forced to undergo unsafe abortion.

## Discussion

The results of this study demonstrate that the issue of induced abortion within the examined articles is framed within two main discourses. One prominent discourse is religious and put forward by representatives of the Catholic movement, emphasizing the sanctity of life of the fetus, i.e., that the life of the fetus stands above everything else. Another discourse focuses on human rights aspects, namely the right to life of the unborn child on the one hand and on the other hand women’s rights to health. Within this discourse legal and medical discourses are also drawn upon. These are for example characterized by arguments referring to legal documents and abortion related experiences from the medical field. Furthermore, the articulation of the discourses has bearing on who is portrayed as to be blamed for and who is the victim of abortions. On the one hand exists the reasoning that women are victims as a result of the strict abortion legislation as it forces them to undergo illegal and unsafe abortions. On the other hand it is argued that the danger consists of that many Ugandan women, as well as the personnel assisting them, break the law by conducting abortions, i.e., committing murder.

This study displays the sensitivity of the topic of abortion within the Ugandan society. Examples of how this delicacy is manifested include the way different actors and their activities are portrayed and presented, in addition to how stakeholders themselves choose to convey their messages through the media. It is also showcased by the nonexistence of certain statements and actors. The key groups described in the examined articles involve church leaders and others acting against abortion in the name of religion, groups looking to change Uganda’s strict legislation on abortion, as well as young women who have undergone abortion. Noticeable is also the complete lack of statements related to the Ugandan abortion legislation by the country’s political leaders.

The strong societal position of the Catholic Church is evident as they are able to set moral standards through a religion based discourse. This discourse determines the immorality of girls and unmarried women being sexually active, the shame of unwanted pregnancies and the need for abortions. Through their rhetoric they are able to produce and reproduce the stigma of abortion [40]. Evident within this discourse is also the absence of statements on men’s role, involvement and responsibility in relation to unintended pregnancies. Furthermore, this is an approach that takes for granted that all women are naturally capable and should be willing to care for their babies, no matter under what circumstances they have gotten pregnant [28]. An underlying problem is the lack of gender equity [26]. The prevailing culture and patriarchal system in Uganda maintain values that privilege men in the allocation of roles and resources. For example do women tend to be poorer and more illiterate than men, and women have fewer contacts in the public sphere [41]. Moreover do these gender structures hinder Ugandan women from being able to negotiate safe sex or contraception [26].

The alternatives to liberalized abortion legislation are framed as good or bad depending on the nature of the article. Described alternatives include how mothers who have wanted to abort can be helped to go through their pregnancies and then feel capable of taking care of their babies. Others include how women’s health as well as the Ugandan health care system is affected negatively as a result of not adopting a more liberal legislation on abortion. Studies have for example shown that cases where women suffer from complications resulting from unsafe abortions are a frequently acknowledged source of stress among midwives in Uganda and that this is explained by the additional workload on an already burdened health care system [42]. Other scenarios portray how newborn babies are abandoned because their mothers did not have access to abortion.

The argumentation questioning the country’s strict abortion law is generally less aggressive than the one against abortion. Argumentation favoring relaxed abortion legislation “problematizes” the strict abortion law and describes its consequences, rather than openly criticizing groups of oppositional views and using strong words. It is evident that these actors, with other views than that of the church, need to be careful in their statements as being seen as going against religion and the will of God would impact negatively on how they are looked upon by the general public, making it harder for them to move forward with their agenda. It is likely that this to a large extent also explains the complete lack of statements related to the Ugandan abortion law by the country’s political leaders within the examined articles. Thus, this absence of certain views and angles from the country’s political arena can also help explain the prevailing social climate and different power relations in Uganda [29]. One example of how groups looking to change the abortion legislation work is to refer to international measures, such as statistics on maternal mortality and morbidity in relation to changed abortion laws in other countries, when arguing for relaxation of the Ugandan abortion laws. Another example is how international conventions signed by Uganda are put forward, stating the obligation to protect women’s rights.

At the same time, however, this development has been used by religious groups in order to describe the increasing immorality of the global society as warning examples of what Uganda needs to keep away from. It is claimed that new laws should not be imposed on Africa by the West.

The stories bringing up individual cases of girls and women who have undergone unsafe abortions might be used in order to deter others from doing the same. This phenomenon has been described as “cautionary tales”, i.e., by using a narrative approach the aim of these stories is to prevent individuals from taking the same actions as the individual(s) featured in the texts [43]. This was for example manifested by the frequent usage of words such as “pain” and “kill” when describing what abortion is all about. On the other hand, it is important to note that reference to the same type of stories has been made, although to a lesser degree, in order to highlight the severe consequences of women seeking unsafe abortions as a result of the existing strict abortion law. In those cases emphasis tends to be put on the stigma of abortion and how girls and women who see themselves forced to undergo abortion are treated disrespectfully.

Previous research suggests that gender equality and the call for improved reproductive rights and change of abortion laws are often preceded by increased social and democratic development [44]. This is something that has shown to both be affected by and affecting levels and scope of women’s rights campaigning [44]. The intensity of debate in any country regarding abortion reforms has also shown to ebb and flow at government level and among the wider public, depending on political administration in power as well as the breadth and success of efforts being made by advocates [6]. Example of such deliberation is the abortion debate that took place in the 1980s in the United States where pro-choice activism was seen as the intrusion of secularism and narcissism by right-to-life protesters [45]. In the same way pro-choice advocates in Uganda today tend to be referred to as an evil power by so called pro-life advocates, aiming at destroying African families and the African society.

Religion, rights and a focus on who is portrayed as a victim and on who is to blame for abortions taking place, seem to be the mainstays surrounding the discourse on the abortion legislation in Uganda. The issue of *gender equality*, however, is only brought up as a background factor and many times as a rather unchangeable condition in the discourse on abortion legislation in Uganda. Nevertheless, gender perspectives have become more and more prominent within the international discourse on health and rights, and the role that gender structures play when it comes to questions related to abortion laws is important to take into account and to scrutinize [28].

Parallel discourses can be an indication of social change [29]. However, although there exist parallel discourses among the material of this study, no evident changes in their contents could be seen during the time period under study, and unlike the discourse on abortion on an international level it did not indicate an increase in the possibility for more scientifically based public debate on the issue. All the same, there is potential for social change in Uganda as several representatives of the Ugandan health care sector argue for abortion rights. Moreover, those who argue against abortion battle the influence of the international community in the form of policy documents and statistics, which can be difficult in the long run.

It is evident that the high prevalence of unsafe abortions in Uganda requires a solution. There is need for a more inclusive and varied debate that highlights and problematizes the current situation, especially from a gender perspective. Furthermore, there is a need for reduction of the stigma surrounding abortion in order to constructively being able to discuss solutions to the many unsafe abortions taking place. This requires collaborative actions including insights on the matter from different angles, including for example the ones of social scientists, theologians, advocates for health, rights and justice, and community members who are affected by abortion stigma [46].

### Methodological considerations

Only written text has been used in this study, and only in English, although the discourse on abortion in Uganda is prevalent also in other forms, forums and languages. The study focuses on the textual contents of the articles rather than on their visual aspects. Nor does it take into consideration who the authors of the articles are and under what category the articles fall, for example if they are defined as national news or if it is a column. However, the approach of this study was chosen as it is believed to be overarching and that the content of the material used reaches various groups of the Ugandan society, directly or indirectly. The reconstruction of the debate content should not be considered as a complete description of it, but it is believed that such an analysis of the conceptual content provides a thorough examination of the focus that the articles have and how abortion related issues are described, formulated and problematized. It specifically examined represented actors within the texts and what stand they take on the issue of abortion or what opinions the authors of the articles convey in terms of ways of describing the actors and events included in the articles. Moreover, the defined relations between the actors, societal structures and the phenomenon of abortion have been examined in order to highlight in what ways these are constructed and reinforced within the chosen media. Measures to increase the trustworthiness of this study include a methodological description, and the interpretation of the data was continuously discussed and re-evaluated by the members of the multidisciplinary research team [47].

## Conclusions

This study displays the sensitivity of the topic of abortion within the Ugandan society, manifested in the way different actors and their activities are portrayed and presented, including how stakeholders choose to convey their messages through the media and showcased by the nonexistence of certain statements and actors. Two main discourses are found; one religious put forward by representatives of the Catholic movement, emphasizing the sanctity of life of the fetus. Another focusing on human rights aspects; the right to life of the unborn child and women’s rights to health respectively, here also legal and medical discourses are drawn upon. The articulation of the discourses has bearing on who is portrayed as to blame for and who is the victim of abortions, thus having implications for the stigma surrounding abortion. To decrease the number of maternal deaths, this study points to the need for a more inclusive and varied debate that problematizes the current situation, especially from a gender perspective.

## References

[1] WHO (2012). Trends in maternal mortality: 1990–2010.

[2] Shah I, Åhman E (2009). Unsafe abortion: Global and regional Incidence, Trends, Consequences, and Challenges. J Obstet Gynaecol Can.

[3] Worell J (Ed.). Encyclopedia of Women and Gender. Sex similarities and differences and the impact of Society on gender. San Diego: Academic Press; 2001.

[4] Singh S, Sedgh G, Hussain R (2010). Unintended Pregnancy: Worldwide Levels, Trends, and Outcomes. Stud Fam Plann.

[5] Atuyambe L, Mirembe F, Johansson A, Kirumira EK, Faxelid E (2005). Experiences of pregnant adolescents–voices from Wakiso district, Uganda. Afr Health Sci.

[6] Singh S, Wulf D, Hussain R, Bankole A, Sedgh G (2009). Abortion Worldwide: A Decade of Uneven Progress.

[7] Tsui AO, Casterline J, Singh S, Bankole A, Moore AM, Omideyi AK, Palomino N, Sathar Z, Juarez F, Shellenberg KM (2011). Managing unplanned pregnancies in five countries: Perspectives on contraception and abortion decisions. Glob Public Health.

[8] Rohlinger D (2006). Friends and foes: Media, politics, and tactics in the abortion war. Soc Probl.

[9] Shellenberg KM, Hessini L, Levandowski BA (2014). Developing a Scale to Measure Stigmatizing Attitudes and Beliefs About Women Who Have Abortions: Results from Ghana and Zambia. Women Health.

[10] Major B, Gramzow RH (1999). Abortion as stigma: Cognitive and emotional implications of concealment. J Pers Soc Psychol.

[11] Hessini L (2014). A Learning Agenda for Abortion Stigma: Recommendations from the Bellagio Expert Group Meeting. Women Health.

[12] Major B, O’Brien LT (2005). The social psychology of stigma. Annu Rev Psychol.

[13] Kate Cockrill K, Hessini L (2014). Introduction: Bringing Abortion Stigma into Focus. Women Health.

[14] Ringheim K, Huntington D, Piet-Pelon NJ (1999). Ethical issues in postabortion care research involving vulnerable subjects. Postabortion Care: Lessons from Operations Research.

[15] WHO. Global Health Observatory. [http://www.who.int/gho/maternal_health/countries/uga.pdf?ua=1] (Retrieved March 31, 2013).

[16] UBOS, ICF International. Uganda Demographic and Health Survey 2011. Kampala, Uganda: UBOS; Calverton, MD, USA: ICF International, 2012.

[17] Khan S, Bradley S, Fishel J, Mishra V (2008). Unmet Need and the Demand for Family Planning in Uganda: Further Analysis of the Uganda Demographic and Health Surveys, 1995–2006.

[18] Nalwadda G, Mirembe F, Tumwesigye NM, Byamugisha J, Faxelid E (2011). Constraints and prospects for contraceptive service provision to young people in Uganda: provider’s perspectives. BMC Health Serv Res.

[19] Singh S, Prada E, Mirembe F, Kiggundu C (2005). The incidence of induced abortion in Uganda. Int Fam Plan Perspect.

[20] Nalwadda G, Nabukere S, Salihu HM (2005). The abortion paradox in Uganda: fertility regulator or cause of maternal mortality. J Obstet Gynaecol.

[21] Center for Reproductive Rights (2011). 10 key points about Uganda’s laws and policies on termination of pregnancy. Fact Sheet.

[22] WHO (2011). Unsafe Abortion: Global and Regional Estimates of the Incidence of Unsafe Abortion and Associated Mortality in 2008.

[23] Guttmacher Institute (2013). Fact sheet. Abortion in Uganda.

[24] Among B. Plans to frame critical church leaders leaked. Daily Monitor. [http://www.monitor.co.ug/News/National/Plans-to-frame-critical-church-leaders-revealed/-/688334/1404538/-/11nloxlz/-/index.html] (Retreived 2012, May 13).

[25] Ninsiima R. Ignorance increasing abortions among adolescents. The Observer. [http://www.observer.ug/index.php?option=com_content&view=article&id=15989:ignorance-increasing-abortions-among-adolescents] (Retreived November 23, 2011).

[26] Sibbald B (2007). Ugandan Government resolves to make safe motherhood a priority. Can Med Assoc J.

[27] York G. Abortion’s veil of silence threatens Ugandan women. The Globe and Mail. [http://www.theglobeandmail.com/life/health-and-fitness/abortions-veil-of-silence-threatens-ugandan-women/article4249025/] (Retrieved 2011, November 6).

[28] Bacchi L (1999). Women, Policy and Politics. The Construction of Policy Problems.

[29] Fairclough N, Wetherell M, Taylor S, Yates SJ (2001). The Discourse of New Labour. Discourse as Data. A guide for analysis.

[30] Wandera D (2010). Bishop demands life jail term for child killers.

[31] Katende MJW (2009). Dare to bear the dear baby.

[32] Lukwago J (2012). Keep away from sex, Lwanga tells youths.

[33] Nalubega F (2010). Legalise abortion to save babies and mothers, says health expert.

[34] Tweheyo R (2010). Increase contraceptive use to reduce maternal deaths.

[35] Emojong JA (2010). Produce more, say anti-abortion activists.

[36] Odeke F (2011). Tororo medic arrested over abortion.

[37] Tumushabe F (2011). To abort or carry the baby?.

[38] Matanda D (2009). A very conflicted discourse on abortion.

[39] Katende C (2010). Hope for baby abandoned in taxi.

[40] Van Dijk TA (1997). Discourse Studies. A multidisciplinary introduction.

[41] Ellis A, Manuel C, Blackden M (2005). Gender and Economic Growth Assessment for Uganda: Unleashing the Power of Women.

[42] Paul M, Gemzell-Danielsson K, Kiggundu C, Namugenyi R, Klingberg-Allvin M (2014). Barriers and facilitators in the provision of post-abortion care at district level in central Uganda – a qualitative study focusing on task sharing between physicians and midwives. BMC Health Serv Res.

[43] Roy SC (2008). ‘Taking charge of your health’: discourses of responsibility in English-Canadian women’s magazines. Sociol Health Illn.

[44] Pillai VK, Gupta R (2011). Reproductive rights approach to reproductive health in developing countries. Glob Health Action.

[45] Ginsburg F. Contested Lives: The Abortion Debate in an American Community. San Diego: University of California Press; 1998.

[46] Cockrill K (2014). Commentary: Imagine a World Without Abortion Stigma. Women Health.

[47] Shenton AK (2004). Strategies for ensuring trustworthiness in qualitative research projects. Educ Inf.

